# Regenerative Potential of Mesenchymal Stem Cells’ (MSCs) Secretome for Liver Fibrosis Therapies

**DOI:** 10.3390/ijms222413292

**Published:** 2021-12-10

**Authors:** Simona-Rebeca Nazarie (Ignat), Sami Gharbia, Anca Hermenean, Sorina Dinescu, Marieta Costache

**Affiliations:** 1 Department of Biochemistry and Molecular Biology, University of Bucharest, 050663 Bucharest, Romania; simona.ignat@unibuc.ro (S.-R.N.); samithgh2@gmail.com (S.G.); anca.hermenean@gmail.com (A.H.); marieta.costache@bio.unibuc.ro (M.C.); 2 “Aurel Ardelean” Institute of Life Sciences, “Vasile Goldiș” Western University of Arad, 310025 Arad, Romania; 3 The Research Institute of the University of Bucharest (ICUB), University of Bucharest, 050663 Bucharest, Romania

**Keywords:** liver fibrosis, cell-free therapy, mesenchymal stem cells, secretome, extracellular vesicles

## Abstract

Chronic liver injuries lead to liver fibrosis and then to end-stage liver cirrhosis. Liver transplantation is often needed as a course of treatment for patients in critical conditions, but limitations associated with transplantation prompted the continuous search for alternative therapeutic strategies. Cell therapy with stem cells has emerged as an attractive option in order to stimulate tissue regeneration and liver repair. Transplanted mesenchymal stem cells (MSCs) could trans-differentiate into hepatocyte-like cells and, moreover, show anti-fibrotic and immunomodulatory effects. However, cell transplantation may lead to some uncontrolled side effects, risks associated with tumorigenesis, and cell rejection. MSCs’ secretome includes a large number of soluble factors and extracellular vesicles (EVs), through which they exert their therapeutic role. This could represent a cell-free strategy, which is safer and more effective than MSC transplantation. In this review, we focus on cell therapies based on MSCs and how the MSCs’ secretome impacts the mechanisms associated with liver diseases. Moreover, we discuss the important therapeutic role of EVs and how their properties could be further used in liver regeneration.

## 1. Introduction

Liver fibrosis is a wound healing response that degenerates, and is characterized by excessive accumulation of extracellular matrix (ECM) components that form scar tissue [1]. There are many causes for liver fibrosis, such as alcohol abuse, non-alcoholic steatohepatitis (NASH), viral or autoimmune hepatitis, non-alcoholic fatty liver disease (NAFLD), ischemic injury, and congenital syndromes [2,3]. Liver fibrosis, left untreated, can progress towards irreversible end-stages of liver failure, liver cirrhosis and hepatocellular carcinoma [4]. Most frequently, either removing the cause or the use of antifibrotic drugs would not be completely effective in treating the disease, and liver transplantation is often recommended [5].

**Figure 1:**
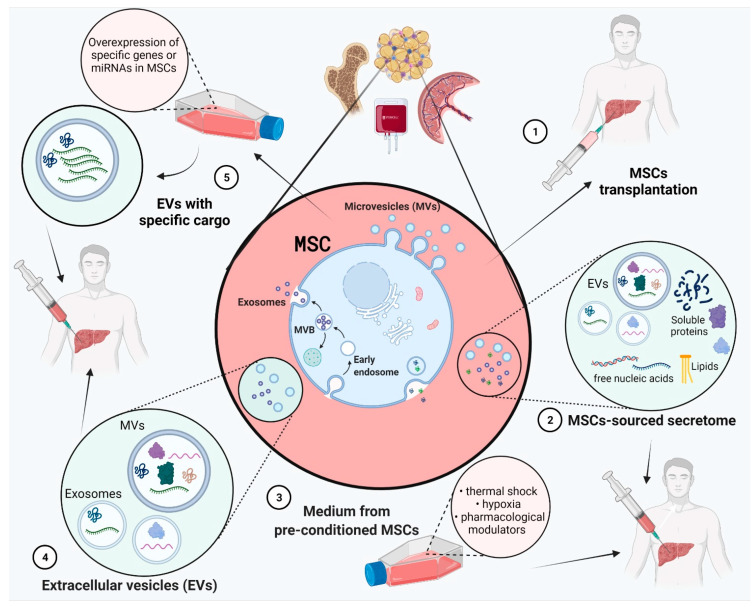
Liver fibrosis therapies based on mesenchymal stem cells (MSCs). MSCs can be isolated from many organs and tissues such as bone marrow, adipose tissue, peripheral blood, placenta, umbilical cord etc. MSC-based therapy strategies: (**1**) MSCs transplantation directly to the patient; (**2**) Use of MSC-sourced secretome (soluble molecules and extracellular vesicles (EVs)); (**3**) Use of medium from pre-conditioned MSCs (physical or environmental shock and pharmacological modulators); (**4**) Use of MSC-sourced EVs; (**5**) Use of MSC-sourced EVs with upregulated expression of genes or miRNAs. Figure created with BioRender.com on 6 December 2021.

Cell therapies have emerged as an alternative to liver transplantation in order to induce tissue regeneration (Figure 1). Numerous cell sources have been investigated for their regenerative potential, either hepatocytes [6], or different types of stem cells [7,8]. There are two major categories of stem cells, embryonic stem cells (ESCs) and adult stem cells (ASCs). Two sources of pluripotent stem cells that can differentiate into hepatocyte-like cells are ESCs, derived from embryos [9,10,11], and induced pluripotent stem cells (iPSCs) [12]. ASCs are multipotent stem cells and have a more limited cell differentiation potential than ESCs. ASCs considered for liver regeneration are liver stem cells (LSCs) [13,14] and mesenchymal stem cells (MSCs) [15,16] from different tissue sources, such as bone marrow (BM-MSCs) [17], adipose tissue (ADSCs) [18,19,20], umbilical cords (UC-SCs), peripheral blood, cartilage, etc. MSCs show low immunogenicity, have a self-renewal ability, are easily obtainable, and can be used without ethical issues, which make them the perfect candidate for liver regeneration [21]. They can migrate to injury sites as a response to cellular damage signals, and they have been shown to promote other cells’ migration to liver sites, differentiate in hepatocyte-like cells and participate in liver regeneration via paracrine mechanisms [22,23,24].

Cell therapies based on MSCs could help liver regeneration directly, by hepatogenic differentiation or cell migration to liver sites, or indirectly, in a paracrine manner via its secretome [23]. MSC-sourced secretome contains many soluble molecules and extracellular vesicles (EVs) that direct tissue repair and regeneration [9,25]. Cell based therapies come with a number of limitations and risks, and cell-free strategies, such as MSC-sourced secretome, could represent an improved alternative. The use of secretome rather than cells avoids the risks associated with tumorigenesis or potential differentiation into pro-fibrotic cells [26,27]. MSC-sourced secretome can be stored over long periods of time without toxic cryopreservative agents and also, it could be produced in large quantities over a short period of time with low costs. Moreover, the content of MSC-sourced secretome could be modified for specific desired therapeutic effects [27]. Cell-based therapies face an additional challenge in regard to its method of transplantation to patients, as such, an effective method has not yet been identified, and cells may end up in other organs [7].

The aim of this review is to present an updated view of cell-based strategies for liver regeneration, with special emphasis on MSC-sourced secretome and derived EVs, and the way they interact with specific pathways involved in liver diseases.

## 2. Liver Fibrosis and Signaling Pathways

Liver fibrosis occurs after complex interplay between different hepatic cells, and many signaling pathways are involved in its progress [5]. Hepatotoxic agents affect the hepatocytes in the liver and induce their apoptosis [28]. Apoptotic bodies and reactive oxygen species (ROS) further activate hepatic stellate cells (HSCs) and stimulate the infiltration of inflammatory cells [28]. Inflammatory cells release a number of inflammatory factors which will maintain high levels of inflammation in the liver and stimulate HSCs to produce collagen [29]. Hepatic macrophages, such as Kupffer cells (KCs) or monocyte-derived, can be classified as pro-inflammatory macrophages (M1) and immunoregulatory macrophages (M2) [30]. They can contribute to the progress of liver fibrosis and maintain the activated state of HSCs [31].

Liver fibrosis is characterized by the high levels of ECM, and most components are produced by activated HSCs [32]. HSCs produce collagen and other ECM proteins in the normal liver as well, but during liver fibrosis, they transdifferentiate towards a myofibroblast-like phenotype [33]. Activated HSCs lose their ability to store vitamin A-droplets, acquire contractile, proinflammatory and fibrogenic properties, and express α-smooth muscle actin (α-SMA) [34,35]. ECM changes its composition in liver injuries, and adds up to six times more than in normal conditions [36]. It is rich in collagens (I, III, IV), fibronectin, vimentin, elastin, and laminin, and metalloproteinases (MMPs) activity is reduced while the expression of tissue inhibitors of MMPs (TIMPs) is upregulated [37,38].

There are several signaling pathways that play an important role in liver fibrosis development, and especially in HSCs activation. Activated KCs and activated HSCs produce a platelet-derived growth factor (PDGF) [39]. This factor can further activate other signaling pathways in HSCs, such as PI3K/Akt, JAK/STAT, and Ras/Raf systems, that will in turn regulate the expression of fibrotic markers (collagen I, MMPs, TIMPs) [40].

One of the most important signaling pathways in the development of liver fibrosis is the transforming growth factor β (TGF-β)/SMAD pathway. TGF-β is produced by macrophages, hepatocytes and even activated HSCs, as a latent precursor which is stored in the ECM [41]. Its activated form is cleaved by proteases and bound to the specific receptors, which will activate the signaling pathway through phosphorylation of SMAD molecules. A protein complex with phosphorylated SMAD2 and SMAD3, translocates in the nucleus and regulates the expression of fibrotic markers [42,43].

Another pathway involved in liver fibrosis is the Wnt/β-catenin signaling pathway. β-catenin is an adhesion molecule and a transcription factor, and its functions are mainly regulated by Wnt proteins [44]. When the pathway is activated, Wnt inhibits β-catenin phosphorylation, which in turn will increase the levels of unphosphorylated β-catenin in the cytoplasm. This will induce its translocation in the nucleus and the transcription of genes such as *Cyclin D1*, *c-Myc*, *Axin-2*, and *c-Jun* [45]. Wnt/β-catenin signaling is upregulated in activated HSC, and it can lead to the overexpression of α-SMA and collagen [46,47].

## 3. Composition of MSCs’ Secretome (Derived Soluble Factors CM/EV)

MSCs secrete a number of molecules, such as soluble proteins, free nucleic acids, lipids and EVs, all accounting for MSCs’ secretome [9,27,48]. MSCs can be isolated from many types of tissues and organs, and even though they share many characteristics and show regenerative potential, their secretome content may vary based on their origin. Differences in MSC-sourced secretome have been pointed out between ADSCs, BM-MSCs and UC-SCs [10,12,49]. All types of MSCs secrete factors such as cytokines, chemokines, growth factors, and anti-inflammatory factors. Overall, it was proved that MSC-sourced secretome shows immunomodulatory, anti-inflammatory and anti-apoptotic activity, regulates angiogenesis, stimulates wound healing and tissue repair, and has antitumor and anti-microbial effects [27]. However, the exact mechanisms through which MSC-sourced secretome exerts its role are not fully understood, and the exact molecules responsible for each effect are not specifically correlated.

MSCs secrete not only a number of anti-inflammatory, but also pro-inflammatory cytokines which mediate its immunomodulatory effect. The secretome contains anti-inflammatory cytokines, such as tumor necrosis factor β1 (TNFβ1), interleukin (IL) 13, IL18 binding protein (IL18BP), ciliary neutrophic factor (CNTF), neurotrophin 3 (NT-3) factor, IL10, IL12p70, IL17E, IL27, IL1. MSCs also secrete pro-inflammatory cytokines, such as IL1b, IL6, IL8 and IL9 [27] and the pleiotropic cytokine transforming growth factor β (TGF-β) [49]. Moreover, MSCs secrete growth factors with regenerative potential, such as vascular endothelial growth factor (VEGF), hepatocyte growth factor-1 (HGF-1), leukemia inhibitory factor (LIF) [49], and keratinocyte growth factor (KGF) [50]. HGF shows anti-fibrotic properties, and can induce apoptosis of activated HSCs and promote hepatocyte proliferation [51,52].

MSC-sourced secretome contains different types of EVs: exosomes (exo), microvesicles (MVs) and apoptotic bodies, classified based on their size and biogenesis, but not on other specific markers [26,53]. Recent studies suggest that MSC-derived EVs, and especially exosomes, play an important role in the therapeutic effect of MSCs through paracrine mechanism. They are easily obtainable through filtering and ultracentrifugation protocols [27,54]. EVs are small particles originated from the plasma membrane, which are secreted from cells, either as part of a disposal mechanisms or as communication mediators between cells [55]. Exosomes are small spherical bilipid membrane vesicles (40–150 nm) which emerge from fusion with early endosomes and form multivesicular endosomes (MVEs). These will fuse with the cell membrane and exosomes will be expelled from the cell [56]. MVs are bigger vesicles (100–1000 nm) formed from direct outward budding of the plasma membrane [26].

Apoptotic bodies (1–5 µm) are formed during the self-destructive actions of a cell during apoptosis, by the outward budding of the plasma membrane. Opposed to exosomes and MVs, apoptotic bodies contain components of cell degradation from dying cells and undergo phagocytosis by macrophages [57].

EVs hold an important role in intercellular communication as their cargo includes proteins, lipids, mRNA, microRNA and they can be safely transferred between cells. Their cargo is determined by their tissue source and influences their effects. Moreover, MSCs can be modified in order to manipulate the EVs cargo to contain specific molecules [26,58,59].

## 4. Role of MSCs-Sourced Secretome in Liver Regeneration

### 4.1. MSCs in Liver Disease Treatment

The practice of MSC transplantation emerged as a way to improve liver function, in terms of bringing an extra source of cells with differentiation potential that would help speed up the regeneration process. In addition, it was known that stem cells-sourced secretome is rich in molecules also involved in regenerative mechanisms.

MSCs could be induced to differentiate into hepatocyte-like cells that can contribute to liver regeneration [21,60]. The source of MSCs greatly influences their differentiation potential and the type of cytokines and growth factors needed to induce it [61]. There is no standard for the growth factor cocktail, which is different based on the MSCs’ source and between different studies. Most of the studies use fibroblast growth factor (FGF) and epidermal growth factor (EGF) in a primary induction step that stimulates the proliferation of MSCs [62]. HGF is a pleiotropic cytokine which regulates proliferation, differentiation and migration of MSCs, and it is frequently used in differentiation studies [63]. Other factors used for differentiation are nicotinamide (NTA) and insulin-transferrin selenium (ITS) which promote the proliferation and survival of primary hepatocytes [64]. Oncostatin M (OSM) and dexamethasone (Dex) also help with the maturation process of developing hepatocytes [65].

However, in many studies, MSCs are not differentiated before being transplanted at the injury site, rather their differentiation is directed in vivo by ECM and interactions with other liver cells [66]. MSCs transplantation has been tried via many routes and in many dosages, which influenced the outcome of each study [67]. MSCs transplantation into the liver has been realized by intravenous, intrahepatic, intraperitoneal, intrasplenic and portal vein injection. Most frequently, the peripheral vein and hepatic artery are used as transplantation routes [21].

The potential of MSC transplantation as an anti-fibrotic therapy was evaluated both in vitro, by establishing co-culture systems between HSCs and MSCs, and in vivo, by an MSC injection to liver fibrosis animal models. In a study by Wang et al. [68], human BM-MSCs were co-cultured with LPS-activated human hepatic stellate cells from the LX2 cell line. Expression of fibrotic markers, such as α-SMA, Col-1, and TLR4, were significantly decreased compared to activated HSCs, suggesting the hBM-MSCs inhibit HSCs’ activation. Moreover, hBM-MSCs inhibited the activation of the NF-kB pathway (reduced the expression of downstream molecules—cyclinD1, c-Myc, Mmp9, CXCR4, Cox2 and VEGF) in LX2 cells. Another study by Lin et al. [69] co-cultured human BM-MSCs and activated human HSCs. They found that BM-MSCs inhibit the proliferation of activated HSCs and promote, instead, their apoptosis by increasing the activity of caspase 3/7.

In a study by Wu et al. [70], AD-MSCs were injected intravenously to mice with CCl_4_-induced liver fibrosis. Up to 3 weeks after transplantation, liver fibrosis was significantly ameliorated and AD-MSCs were proven to migrate, survive and differentiate into hepatic cells. MSCs isolated from BM were also proven to positively influence liver regeneration in rats with thioacetamide-induced liver fibrosis. BM-MSCs were injected intraperitoneally and, for up to 6 weeks, the number of inflammatory cells was reduced and liver function improved [71]. In another study by Hao et al. [72], the ability to attenuate liver fibrosis was investigated in BM-MSCs and AD-MSCs, both in vitro and in vivo. In the in vitro co-culture system, AD-MSCs proved to be more efficient than BM-MSCs in inhibiting proliferation and activation of HSCs, and also in promoting HSCs apoptosis. For the in vivo evaluation, the two types of MSCs were injected through the portal vein twice in 4 weeks in rats with CCl_4_-induced liver fibrosis. Inflammation and fibrosis were reduced in a similar manner after treatment with either AD-MSCs or BM-MSCs.

Furthermore, clinical trials have established positive roles of MSCs transplantation to patients with chronic liver conditions [73,74,75,76]. Autologous BM-MSCs transplantation was performed for alcohol-related liver cirrhosis via arterial injections. The patient groups that received BM-MSCs showed improved reductions in the proportion of collagen, as an indicator of fibrosis, and Child-Pugh scores were significantly improved as well [75]. In another study by Jang et al. [73], autologous BM-MSCs were amplified for one month and then injected via the hepatic artery to patients with alcoholic cirrhosis. The Child-Pugh score was improved after transplantation, and also gene expression levels of *TGF-β1*, *collagen type I* and *α-SMA* were significantly decreased.

Nonetheless, there are other clinical studies that show no improvement in liver function after MSC transplantation [77,78]. More extensive studies are needed to confirm the safety and efficiency of MSC transplantation to patients with chronic liver injuries.

Many studies indicated that hepatocytes differentiated from MSC represent only ~ 1% of the total liver mass after transplantation [79]. Therefore, the focus switched from the efficiency in terms of MSCs differentiation and MSCs transplantation to the overall effect MSC-sourced secretome.

### 4.2. Effect of MSC-Derived Conditioned Medium (MSC-CM)

The conditioned medium (CM) from MSCs contains all the components of the secretome, and is easily obtainable from cultured MSCs by centrifugation and filtration [27]. CM was frequently used in studies to evaluate the effects of MSC-sourced secretome in liver regeneration, without the risks associated with cell transplantation.

UC-MSCs were isolated and differentiated in vitro into hepatocyte-like cells, and their secretome was obtained in a study by An et al. [80]. CM-MSCs, from differentiated and undifferentiated UC-MSCs, were distributed to TGF-β1-activated HSCs and mice with thioacetamide (TAA) and CCl_4_-induced liver fibrosis. CM-MSCs inhibited HSCs activation and reduced the expression of fibrotic factors such as α-SMA, collagens, metalloproteinases, TGFβ, and Smad proteins in the TGFβ signaling pathway. One highly expressed protein was identified in the UC-MSCs secretome, milk fat globule-EGF factor 8 (MFGE8), and it was proven, in the study, to show anti-fibrotic action similar to the effect of CM-MSC.

In a study by Pinheiro et al. [81], CM from AD-MSCs was injected to mice with cholestatic liver fibrosis induced by bile duct ligation. CM-MSC treatment decreased levels of hepatic enzymes and collagen deposition in the liver, and pro-fibrotic IL-17A was decreased and IL-6 and IL-4 were increased. Similarly, CM from BM-MSCs injected into mice with CCl_4_-induced liver fibrosis, led to an immunosuppressive response, correlated with the upregulation of M2-type macrophages which will secrete anti-inflammatory molecules such as CCL-1 and IL-10. Moreover, CM-MSC stimulated the liver repair system, inhibited inflammatory infiltration and promoted the apoptosis of activated HSCs [82].

A specific interest in some studies was focused on determining the role of some factors from the MSCs secretome. One such factor is HGF, a potent growth factor involved in liver regeneration [83]. One study co-cultured HSCs with BM-MSCs and identified MSC-secreted HGF to be responsible for suppressing HSCs activation [68]. Furthermore, another study by Yin et al. [84] showed that HGF-transfected hUC-MSCs co-cultured with HSCs promoted HSCs apoptosis and inhibited their activation. Moreover, they downregulated the expression of molecules involved in the TGF-β1/SMAD pathway, such as collagen I, collagen III, TGF-β1, Smad2 and Smad3.

### 4.3. Pre-Treatment of MSCs for Improved Secretome Content

Therapeutic effects of MSCs can be improved by changing the cell culture conditions of MSCs that will further modulate the content of MSCs secretome. Preconditioning can prepare the cells for transplantation in vivo and improve their survival rate and its paracrine effects [85]. Some pro-survival strategies include methods in which cells are exposed to physical or environmental shocks and pharmacological modulators of targeted molecules. One strategy is a thermal preconditioning method at 42 °C for 1–2 h before transplantation, proven to promote cell survival in vivo [86]. Next, hypoxic preconditioning mimics the natural in vivo conditions (1–12% in vivo compared to 21% in vitro) and improves MSCs multipotency and self-renewal abilities [87,88].

Other cell culture conditions can modulate the immunomodulatory properties of MSCs. LPS is a representative Toll-like receptor 4 (TLR4) agonist and can induce MSCs to exert an immunosuppressive phenotype, necessary in order to reduce liver fibrosis-related inflammation [89,90]. In a study by Lee et al. [91], AD-MSCs were pre-cultured for 24 h with a low dose of lipopolysaccharide (LPS). LPS preconditioning of AD-MSCs upregulated the expression of inflammation-related mediators such as IL-6, TNF-α, HGF, and VEGF. CM from LPS-preconditioned AD-MSCs was injected to partially hepatectomized mice, and liver regeneration was found to be improved compared to the control. It was proposed that pro-regenerative effects of the LPS-preconditioned CM were mediated through IL-6/STAT3 activation.

### 4.4. Effect of EVs

A lot of the recent interest has been focused on the EVs in the MSCs-sourced secretome and their specific role in modulating liver repair (Table 1). It was shown that MSC-EVs modulate liver regeneration by regulating many pathways involved in liver fibrosis development. These effects were confirmed on EVs from different types of MSCs derived from adipose tissue, bone marrow, umbilical cord blood, placenta or amnion.

**Figure 2:**
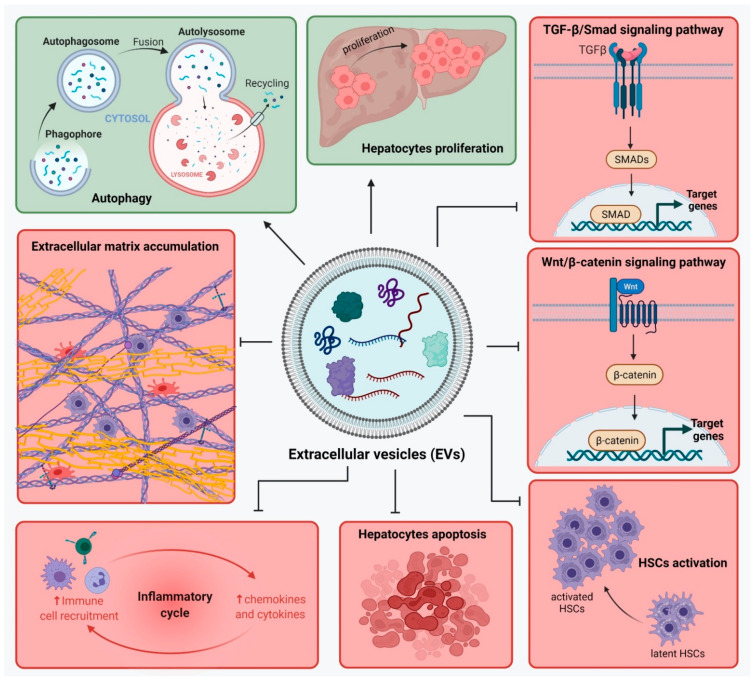
Extracellular vesicles from mesenchymal stem cells (MSC-EVs) modulate liver regeneration by regulating pathways involved in liver fibrosis development. EVs’ content is rich in proteins, lipids, mRNA and microRNA. MSC-EVs may act in two distinct ways: **Green**—Inhibition of: TGF-β1/Smad signaling pathway, Wnt/β-catenin pathway, hepatic stellate cells (HSCs) activation, hepatocyte apoptosis, inflammatory factors secretion, collagen deposition; **Red**—Activation of: autophagy and hepatocyte proliferation. Figure created with BioRender.com on 17 November 2021.

MSC-EVs act upon the TGF-β1/Smad signaling pathway [92,93] and Wnt/β-catenin pathway [94,95], stimulate autophagy, inhibit hepatocyte apoptosis [96], reduce collagen deposition [92,94,97], inhibit activation of HSCs [98], modulate the inflammation by reducing levels of inflammatory factors [99,100,101], and inhibit activation of NLRP3 inflammasome [102] (Figure 2).

One of the first effects of MSC-EVs studied is on HSCs activation [98], which is inhibited. Additionally, MSC-EVs downregulate the expression of fibrotic markers, such as α-SMA, collagen I, collagen III, TGF-β [92,94,97]. In a study by Fiore et al. [93], rat CFSC-2G hepatic stellate cells and mice with TAA-induced liver fibrosis were treated with EVs isolated from hUC-MSCs. EVs from hUC-MSCs inhibited HSCs’ activation in vitro by reducing the expression of α-SMA, collagen I and TGF-β1. The same results were observed in the in vivo model of TAA liver fibrosis-induced BALB/c mice, with significant downregulation of the fibrotic markers’ expression. Other studies used exosomes isolated from hUC-MSCs and treated animal models with CCl_4_-induced liver fibrosis [92,96,97]. They acted upon collagen, reducing its expression. Moreover, expression of α-SMA was reduced in mice and rats with CCl_4_-induced liver fibrosis [92,96], and the TGF-β1/Smad signaling pathway was inhibited in mice with CCl_4_-induced liver fibrosis [97]. EVs from amnion derived MSCs (AMSCs) were used on rat HSCs and rats with CCl_4_-induced liver fibrosis, and successfully reduced expression of α-SMA, collagen I, and timp 1, and stimulated the expression of mmp2 [100]. Other studies with EVs from MSCs, used in vitro or in vivo, also report reduced collagen levels and attenuated HSC activation in liver fibrosis models of CCl_4_-induced Sprague-Dawley albino rats and *S. japonicum*-infected mice [98,103].

Another effect of MSC-EVs is to inhibit hepatocyte apoptosis [96], and instead promote their proliferation [101]. Exosomes isolated from hUC-MSCs were used to treat rats with CCl_4_-induced liver fibrosis. The expression of caspase-3 and Bax was significantly reduced, and the expression of Bcl-2 was increased, which suggest that the apoptosis of hepatocytes, usually associated with liver fibrosis, was inhibited [96]. In another in vivo study on BALB/c mice with liver fibrosis induced by CCl_4_, exosomes from hUC-MSC inhibited hepatocyte apoptosis by reducing the expression of activated caspase 3, and Bax and 8-Oxo-2’-deoxyguanosine (8-OHdG) production [97].

Moreover, EVs derived from MSCs can modulate the expression of Wnt/β-catenin pathway components. Exosomes derived from BM-MSCs inhibited the expression of PPARγ, Wnt3a, Wnt10b, β-catenin, WISP1, and Cyclin D1 in a rat model with CCl_4_-induced liver fibrosis [94]. MSC-EVs can also reduce inflammation by suppressing the infiltration of inflammatory cells [97] and downregulating pro-inflammatory cytokines [99,100,101]. In Kupffer cells, the expression of inflammatory cytokines, such as Tnf-α, Il-1β, and Mcp-1, was downregulated after treatment with EVs isolated from AMSCs [100]. In addition, exosomes from hUC-MSCs reduced the infiltration of inflammatory cells in mice with CCl_4_-induced liver fibrosis [97]. In an extensive study by Jin et al. [101], EVs were isolated from hAD-MSCs and used to treat rats with D-aminogalactose (GalN)-induced acute liver failure (ALF). AD-MSC-EVs significantly influenced the expression level of inflammatory mediators by reducing the expression of IL-1ra, IL-1α, IL-1β, IL-6 and IL-17. They also downregulated the expression of chemotactic factors such as CCL20, CINC-1, CINC-2α/β, CINC-3, CNTF, CX3CL1, CXCL7, CXCL9, CXCL10 and LECAM-1.

**Table 1:** Effects of extracellular vesicles from mesenchymal stem cells (MSC-EVs) in chronic liver injury models in vitro and in vivo.

Source of EV	Type of EV	Liver injury Model	Mechanism of Action	References
hAD-MSCs	Exosomes with miR-122	LX2 cell line	Down-regulated the expression of miR-122 target genes (*P4HA1*, *IGF1R* and *CCNG1*) which are involved in the proliferation and collagen maturation of HSCs	[104]
hUC-MSCs	EVs	LX2 cell line	Suppressed HSCs proliferation and activation	[103]
Amnion-MSCs	EVs	Rat HSCs and KCs activated with LPS	Inhibits HSCs activation (reduced expression of α-SMA, collagen I, increased expression of mmp-2)Downregulated the expression levels of inflammatory cytokines (Tnf-α, Il-1β, and Mcp-1) in KC	[100]
hUC-MSCs	EVs with Insulin Growth Factorlike-I (IGF-I)	Rat CFSC-2G hepatic stellate cell line	Reduced the expression of fibrotic markers (collagen I, α-SMA and TGF-β1, and of pro-inflammatory cytokines IL-6 and TNF-α	[93]
hAD-MSCs	Exosomes with miR-181-5p	Mouse HSCs (HST-T6)	Inhibited HSCs activation by downregulating the expression of miR-181-5p target genes (*Stat3* and *Bcl-2*) and activated autophagy (upregulation of Beclin1)	[99]
Amnion-MSCs	EVs	Rats with CCl_4_-induced liver fibrosis	Reduced expression of α-SMA and attenuated formation of fibrotic septa and pseudolobules	[100]
Rat BM-MSCs	EVs	Rats with CCl_4_-induced liver fibrosis	Reduced collagen deposition and attenuated HSC activation	[98]
hBM-MSCs	Exosomes	Rats with CCl_4_-induced liver fibrosis	Inhibited the expression of Wnt/β-catenin pathway components (PPARγ, Wnt3a, Wnt10b, β-catenin, WISP1, Cyclin D1), α-SMA, and collagen I	[94]
hUC-MSCs	Exosomes	Mice with CCl_4_-induced liver fibrosis	Reduced the expression of collagen I and III, inhibited TGF-β1/Smad signaling pathway and epithelial-to-mesenchymal transition (EMT)	[92]
hUC-MSCs	Exosomes	Mice with CCl_4_-induced liver fibrosis	Reduced oxidative stress, decreased TGF-β levels, and inhibited hepatocyte apoptosis and infiltration of inflammatory cells	[97]
hUC-MSCs	Exosomes	Rats with CCl_4_-induced liver fibrosis	Reduced collagen accumulation and reduced α-SMA and collagen I expression, inhibited inflammation, apoptosis, caspase-3 and Bax expression, and increased Bcl-2 expression	[96]
hUC-MSCs	EVs with Insulin Growth Factorlike-I (IGF-I)	Mice with TAA-induced liver fibrosis	Reduced the expression of collagen I, α-SMA and the profibrogenic cytokine TGF-β1	[93]
hAD-MSCs	EVs with lncRNA-H19	D-aminogalactose (GalN)-induced ALF	Downregulated the expression of inflammatory mediators (IL-1ra, IL-1α, IL-1β, IL-6 and IL-17) and chemotactic factors (CCL20, CINC-1, CINC-2α/β, CINC-3, CNTF, CX3CL1, CXCL7, CXCL9, CXCL10 and LECAM-1), inhibited tissue necrosis, promoted hepatocyte proliferation	[101]
AD-MSCs	Exosomes with miR-17	Mice with LPS/GalN-induced ALF	miR-17 from exosomes inhibited NLRP3 inflammasome activation by targeting TXNIIP in hepatic macrophages	[102]
hUC-MSCs	Exosomes with upregulated miR-145-5p	Rats with CCl_4_-induced liver fibrosis	Inhibited the process of liver fibrosis via miR-145-5p-mediated fascin actin-bundling protein 1 (FSCN1) downregulation	[96]
AD-MSCs	Exosomes with overexpressed mmu_circ_0000623	Mice with CCl_4_-induced liver fibrosis	Regulated autophagy mediated by miR-125/ATG4D, inhibited α-SMA expression	[105]
hAD-MSCs	Exosomes with miR-122	Mice with CCl_4_-induced liver fibrosis	Reduced the expression of TGF-β1 and α-SMA and suppressed the serum levels of HA, P-III-P, ALT, AST and liver hydroxyproline content	[104]
hAD-MSCs	Exosomes with miR-181-5p	Mice with CCl_4_-induced liver fibrosis	Downregulated expression of fibrotic markers (collagen I, vimentin, α-SMA and fibronectin) and of pro-inflammatory factors (TNFa, IL-6 and IL-17)	[99]

### 4.5. Exosomes from MSCs with Specific Overexpressed Cargo Such as miRNAs

An increasing number of studies report enhancing the expression of certain therapeutic genes or miRNAs in MSCs, in order to modulate the cargo of EVs. In addition, the overexpression of specific proteins or miRNAs offers the basis to study their way of action in liver regeneration and how they interact with molecules in signaling pathways.

The expression of miR-122 is reduced in advanced liver diseases, such as cirrhosis, and it is a molecule associated with anti-fibrotic potential [106,107]. MiR-122 was overexpressed in AD-MSCs by using a lentivirus (LV)-mediated transfer of pre-miR-122 precursor molecules (LV-miR-122), and exosomes were isolated from the supernatant of the cells using ExoQuick-TC Kit [104]. Exosomes from miR-122-modified AD-MSCs specifically downregulated, in the LX2 HSC cell line, the expression of miR-122 target genes *P4HA1*, *IGF1R* and CCNG1, which are involved in HSCs activation and proliferation. Moreover, these exosomes with miR-122 acted in vivo in mice with CCl_4_-induced liver fibrosis, and reduced the expression of TGF-β1 and α-SMA, and suppressed the serum levels of HA, P-III-P, ALT, AST and liver hydroxyproline content.

The effect of miR-181-5p is associated with maintaining an undifferentiated state of hepatic progenitor cells, and was investigated in another study [99]. AD-MSCs were transfected with a plasmid encoding miR-181-5p and their exosomes were isolated with ExoQuick-TC Kit. Exosomes from miR-181-5p-modified AD-MSCs suppressed HSCs’ activation in vitro in a mouse cell line, through direct targeting of Bcl-2 and STAT3, and activated autophagy by upregulating the expression of Beclin1. AD-MSCsExo also inhibited the expression of collagen I, vimentin, α-SMA and fibronectin, and of the pro-inflammatory factors, TNFa, IL-6 and IL-17.

Other studies investigated the role of miR-17 and miR-145-5p from exosomes, which are part of MSC-sourced secretome. miR-17 from AD-MSC-Exo was found to inhibit NLRP3 inflammasome activation by targeting TXNIIP in mice with LPS/GalN-induced ALF [102]. miR-145-5p from UC-MSC-Exo was found to attenuate liver fibrosis by downregulation of fascin actin-bundling protein 1 (FSCN1) [96].

EVs also contain long non-coding RNAs that play significant roles in modulating the liver fibrosis mechanisms. In a study by Jin et al. [101], AD-MSCs were induced to overexpress lncRNA H19. They discovered that these EVs can enhance the hepatocyte proliferation and inhibit hepatocyte apoptosis in rats with GalN-induced ALF. They also observed the upregulation of the HGF/c-Met pathway and other downstream pathways, such as PI3K/AKT and STAT3, after treatment with the lncRNA H19-enriched EVs.

## 5. Conclusions

Liver fibrosis is a result of liver injury and an altered wound healing response, which leads to ECM proteins’ accumulation. There are complex interactions between liver cells in liver fibrosis development and many signaling pathways are involved in maintaining the inflammatory state. Therapies for liver fibrosis are constantly under investigation, and the use of strategies based on MSCs show great promise. MSC transplantation showed to improve liver function as it brings a source of cells with differentiation potential towards hepatocyte-like cells. In addition to contributing in restoring the number of hepatocytes in the injured liver, MSC-sourced secretome is rich in molecules involved in regenerative mechanisms by action on liver cells such as hepatocytes, HSCs and macrophages. MSC-secretome acts upon hepatocytes and promotes their survival and proliferation. Especially when MSC-EVs were used, the expression of apoptotic markers was inhibited. In addition, MSCs show immunomodulatory and anti-inflammatory activity though the action of their secretome. They stimulate the production of M2-type macrophages, which will secrete anti-inflammatory molecules. Moreover, MSC-secretome inhibits the infiltration of inflammatory cells and suppresses the expression of inflammatory cytokines in KCs. Activated HSCs represent another important cellular target of the MSC-sourced secretome. Not only does it help reduce their number in the fibrotic liver by stimulating their apoptosis, but it also further inhibits their activation and proliferation, thus helping control the overall pool of HSCs. HSCs are also responsible for the ECM production in liver fibrosis, and MSC-secretome inhibits the production of most ECM proteins and fibrotic markers. In such a manner, MSCs contribute in different ways (direct MSC transplantation, MSC-sourced secretome, CM from pre-treated MSCs, MSC-EVs) to alleviate liver damage and promote tissue regeneration. Cell-free therapies based on MSC-sourced secretome help overcome a number of limitations and risks associated with MSCs transplantation. The use of secretome could mimic the anti-fibrotic effects observed after MSCs transplantation. MSCs secretome is rich not only in cytokines, chemokines, growth factors, and anti-inflammatory factors, but in EVs as well. The use of MSCs-EVs as a treatment for liver fibrosis may be more effective than MSCs therapy, as they can pass through biological barriers and deliver their anti-fibrotic cargo to specific target cells. MSC-EVs were shown to regulate signaling pathways such as TGF-β1/Smad and Wnt/β-catenin, stimulate autophagy and hepatocyte apoptosis, reduce collagen deposition, inhibit activation of HSCs, and modulate the inflammation. Moreover, MSC-EVs cargo can be modified in order to deliver specific proteins of miRNAs with anti-fibrotic properties. So far, these strategies show great promise, but more research is needed to confirm their efficiency and safety, especially for the use of EVs with modified cargo.

## Data Availability

Not applicable.
